# High Lysyl Oxidase (LOX) in the Non-Malignant Prostate Epithelium Predicts a Poor Outcome in Prostate Cancer Patient Managed by Watchful Waiting

**DOI:** 10.1371/journal.pone.0140985

**Published:** 2015-10-26

**Authors:** Maria Nilsson, Christina Hägglöf, Peter Hammarsten, Elin Thysell, Pär Stattin, Lars Egevad, Torvald Granfors, Emma Jernberg, Pernilla Wikstrom, Sofia Halin Bergström, Anders Bergh

**Affiliations:** 1 Department of Medical Biosciences, Pathology, Umeå University, Umeå, Sweden; 2 Department of Surgical and Perioperative Sciences, Urology, Umeå University, Umeå, Sweden; 3 Department of Oncology-Pathology, Karolinska University Hospital, Stockholm, Sweden; 4 Department of Urology, Central Hospital Västerås, Västerås, Sweden; University of Kentucky College of Medicine, UNITED STATES

## Abstract

Lysyl oxidase (LOX) has been shown to both promote and suppress tumor progression, but its role in prostate cancer is largely unknown. LOX immunoreactivity was scored in prostate tumor epithelium, tumor stroma and in the tumor-adjacent non-malignant prostate epithelium and stroma. LOX scores in tumor and non-malignant prostate tissues were then examined for possible associations with clinical characteristics and survival in a historical cohort of men that were diagnosed with prostate cancer at transurethral resection and followed by watchful waiting. Men with a low LOX score in the non-malignant prostate epithelium had significantly longer cancer specific survival than men with a high score. Furthermore, LOX score in non-malignant prostate epithelium remained prognostic in a multivariable analysis including Gleason score. LOX score in prostate tumor epithelium positively correlated to Gleason score and metastases but was not associated with cancer survival. LOX score in tumor and non-malignant prostate stroma appeared unrelated to these tumor characteristics. In radical prostatectomy specimens, LOX immune-staining corresponded to LOX in-situ hybridization and LOX mRNA levels were found to be similar between tumor and adjacent non-malignant areas, but significantly increased in bone metastases samples. LOX levels both in tumors and in the surrounding tumor-bearing organ are apparently related to prostate cancer aggressiveness.

## Introduction

Prostate tumors are generally multifocal and heterogeneous and their natural history varies from harmless to lethal. Current imaging methods cannot sufficiently detect prostate tumors, instead multiple needle biopsies, sampling less than 1% of the prostate volume, are taken from the prostate to identify malignant tumor tissue. Tumor-containing biopsies, hopefully representing the most malignant tumor present, are histologically scored (Gleason score, GS) and together with serum levels of prostate-specific antigen (PSA) used to prognosticate tumor behavior. In the majority of prostate cancer patients these methods have limited ability to predict disease aggressiveness, and better prognostic markers are therefore urgently needed [1].

Non-malignant prostate tissue is always sampled by the biopsies, and recent studies by us and others have suggested that the tumor-bearing organ is differently affected/tinted by indolent vs. aggressive tumors [2]. Changes in the normal parts of the tumor-bearing prostate could in addition to adaptive changes also be the result of field cancerization [3, 4]. Alterations in the non-malignant parts of the tumor-bearing prostate (whatever the cause) are important to identify and validate as they could potentially serve as novel markers to diagnose and prognosticate prostate cancer [2–4]. We have named the tumor-adjacent adapted non-malignant prostate tissue as Tumor Instructed (and thus indicating) Normal Tissue (TINT)[2]. TINT includes morphologically normal appearing epithelium and stroma of the tumor-bearing organ and is not in direct contact with the tumor epithelium and is thus separate from the tumor stroma/tumor microenvironment.

We have previously shown that alterations in prostate TINT epithelium and stroma are related to prognostically important tumor characteristics such as GS and tumor stage, and can be used to evaluate the risk of death from prostate cancer in a watchful waiting cohort [2, 5–13]. In order to explore the TINT concept in more detail we have used an animal model where rat prostate cancer cells are implanted into the prostate of immunocompetent rats. This results in adaptive tumor promoting changes in the tumor-bearing prostate lobe, for instance increased densities of macrophages and mast cells, and increased hyaluronan [8, 9, 14–16]. The nature and magnitude of these changes are related not only to tumor size but also to tumor cell aggressiveness (Adamo et al., unpublished). Gene-expression studies showed that one factor markedly up-regulated in rat TINT was Lysyl oxidase (LOX) [17].

LOX is a secreted copper-dependent amine oxidase, with the primary function to cross-link collagen and elastin (both up-regulated in rat TINT [17]), in the extracellular matrix (ECM). Increased extracellular LOX activity therefore results in a stiffer microenvironment that promotes tumor progression, metastasis, and invasion [18–20]. LOX has also been shown to be required for pre-metastatic niche formation [21]. Recently, LOX, secreted by tumor epithelial cells, was also shown to induce pre-metastatic bone lesions that precedes and facilitates the formation of breast cancer metastases [22]. Increased LOX expression has been associated with a worse outcome in patients with astrocytomas, non-small lung cancer and gastric cancer [23–25]. However, LOX has also been shown to have anti-tumor activity in several cancer types including lung, pancreatic, gastric cancer and nasopharyngeal carcinoma [26–28]. LOX inhibits HRAS-induced tumor formation and reverse HRAS-transformation of fibroblasts [29, 30]. The by-product produced upon cleavage of the LOX proenzyme has also been shown to have tumor suppressive properties [31, 32]. Decreased LOX expression has been observed in a number of cancers and has implicated LOX as a tumor suppressor gene [33].

The role of LOX in prostate cancer is not established and both inhibitory and stimulatory effects are reported. High LOX mRNA expression is associated with high-grade prostate tumors and tumor recurrence [34] and has been shown to correlate with GS [35]. Inversely, LOX mRNA expression was decreased in metastatic compared to primary prostate tumors [36] and low levels of LOX expression have also been reported in high-grade tumors [37, 38], suggesting loss of LOX expression during prostate tumor progression.

The aim of this study was therefore to investigate LOX protein expression in prostate tumor epithelial cells, tumor stroma, tumor-adjacent non-malignant prostate epithelium (TINT epithelium) and in tumor-adjacent non-malignant tumor stroma (TINT stroma) and relate LOX expression in the different compartments to histopathological and clinical parameters as well as to outcome in a historical cohort of prostate cancer managed by watchful waiting (see [12] for details). In summary we find that LOX staining score in prostate TINT epithelium predicted prostate cancer specific survival and gave additive prognostic information to GS.

## Materials and Methods

### Patients

LOX staining score (see below) was analyzed in tissue specimens collected from men who underwent transurethral resection of the prostate (TURP) between 1975 and 1991 and where histological analysis showed presence of prostate cancer (for details see [7]). The study includes 351 men of which 304 (including 265 scored for LOX) men had not received any anticancer treatment prior to the TURP and were followed with watchful waiting. The mean age at diagnosis was 74 years (51 to 95 years) and the median overall follow-up period was 5.2 years (0 to 25.5 years). Local tumor stage was determined at the time of surgery by digital rectal examination and the presence of metastasis was evaluated by radio-nucleotide bone scan. No lymph node staging was performed and serum PSA was not available as the material was collected prior to the PSA era. From the tissue specimens collected we constructed tissue microarrays (TMA) that contained 5–8 samples of tumor tissue (each sample containing both tumor epithelium and tumor stroma) and 4 samples of non-malignant tissue (each sample containing both TINT epithelium and TINT stroma) from each patient [7]. GS was re-assessed when the TMA was constructed and the material has previously been analyzed for factors of potential prognostic significance such as tumor volume, phosphorylated AKT (pAKT) [6], hyaluronan [9], phosphorylated epidermal growth factor receptor (pEGFR) [7], vascular density, tumor cell proliferation [10] and accumulation of different types of inflammatory cells [8, 12] and these data were now related to the current LOX findings. The material was collected according to Swedish regulations at a time when informed consent was not required. The research ethical committee at Umeå university hospital (Regional Ethical Review Board in Umeå) approved of the study and waived the need for consent. Patient information was anonymized and de-identified prior to analysis.

In addition, tissue sections from 10 prostate cancer patients who underwent radical prostatectomy in 2012 were examined for LOX protein and mRNA expression using immunohistochemistry and *in situ* hybridization (see next section). This study was approved by the Regional Ethical Review Board in Umeå, and all patients provided written informed consent.

### LOX immunohistochemistry, and *in situ* hybridization, and mRNA expression

The TMA and radical prostatectomy specimens were stained for LOX using the automatic staining system Ventana Benchmark Ultra (Ventana Medical systems Inc.). Briefly, four μm thick paraffin sections were pretreated with CC1 (Tris/Borate/EDTA Buffer (pH8.0), 64min, 95°C) for antigen retrieval and stained with primary rabbit polyclonal antibody against LOX (Novus Biologicals; Littleton CO, USA, NB100-2530, diluted 1:400). Samples were visualized using ultraView Universal DAB detection Kit (Ventana, Tuscon AZ, USA). Human placenta was used as a positive control for LOX, showing positive staining in decidua and trophoblast cells. Sections were digitized and analyzed using Pannoramic viewer 1.15.2 (3DHistech, Budapest, Hungary).

The *in situ* hybridization for LOX mRNA detection was performed manually using RNAscope 2.0 FFPE Reagent Kit (Advanced Cell Diagnostics Inc., Hayward, CA, USA). Briefly, four μm thick FFPE tissue sections were deparaffinized and pretreated with heat and protease followed by incubation with the probe targeting Hs-LOX for 2h at 40°C. Preamplifier, amplifier, and HRP-labeled oligonucleotides were then hybridized sequentially, followed by chromogenic precipitation development with DAB. Finally, samples were counterstained with Meyers hematoxylin, and analyzed in a bright field microscope. Each sample was quality controlled for RNA integrity with an RNAscope probe for cyclophilin B (PPIB) RNA (positive control) and for nonspecific background with a probe for bacterial dapB RNA (negative control). Specific RNA staining signal was identified as brown, punctuate dots.

Data corresponding to LOX mRNA levels in paired samples of primary prostate tumor and adjacent non-malignant prostate tissue (n = 12), and in castration-resistant prostate cancer (CRPC) bone metastases samples (n = 30) were extracted from previously published studies were the human HT12-v3 Illumina Beadchip gene expression array was used according to the manufacturer’s protocol (Illumina, San Diego, CA, USA) and as previously described [39, 40].

### Scoring of LOX immunoreactivity and *in situ* hybridization

Tumor epithelium, tumor stroma, TINT epithelium and TINT stroma were identified by morphological appearance, and LOX staining intensity and spread was scored separately in these four different compartments as negative (0), weak (1), moderate (2), or strong (3). In the TMA, LOX scores are the median values of five to eight scored samples of tumor tissue or four scored samples of non-malignant tissue for each patient. The immunoreactivity (LOX IR) was scored independently by two different investigators (C. H., 351 patients and M. N., 50 patients) with no knowledge of the patient data. LOX staining was observed in the cytoplasm and often also in nuclei but these two compartments were not scored separately.

In addition, LOX IR and LOX mRNA expression was scored as described above on whole sections from 10 radical prostatectomy samples, including both tumor- and non-malignant epithelium and stroma.

### Hypoxia treatment *in vitro*, RNA Preparation and Quantitative RT-PCR Analysis

Prostate epithelial cells (RWPE-1) and stromal myofibroblasts (WMPY-1, (ATCC, Manassas VA, USA) derived from the peripheral zone of the histologically normal human prostate were cultured according to manufacturer’s protocol (ATCC). The cells were grown in 6 well plates at 37°C for 24 hours in a hypoxia (1.0% O_2_, 5% CO_2_, 94% N_2_) or in normoxia (21.0% O_2_, 5% CO_2_, 74% N_2_). Six replicates were used when total RNA was prepared using the TRIzol method according to protocol (Invitrogen). Total RNA was DNase-treated (DNase 1, Ambion) to remove contaminating DNA, and 0.8 μg was used for synthesis of cDNA according to the manufacturer’s instructions using Superscript II (Invitrogen). The real-time qRT-PCR was performed using Applied Biosystems 7900HT Real-Time PCR System and Taqman Gene Expression Assay (Applied Biosystems). The quantification of mRNA levels was executed in a 20-μl reaction volume with 20 ng cDNA per reaction for LOX (commercially available primer and probe mix, LOX, Hs00942480_m1, Applied Biosystems). Negative controls were run in parallel, and the relative values for each gene were normalized using h18S, (Hs99999901_s1) as reference gene and analyzed in Taqman Analysis Software SDS2.4 (Applied Biosystems).

### Statistics

Bivariate correlations were calculated using the Spearman’s rank correlation test. Correlations between nominal variables and continuous variables were analyzed using the Kendall’s tau b correlation. Data was collected at the time of prostate cancer diagnosis and include all patients in the study. Patients followed with watchful waiting were included in Kaplan-Meier survival analyses and Cox regression analysis. The duration of event-free survival (EFS) is defined as the time from TURP until the date of prostate cancer death, death of other causes, or if no death occurred, until the date of last follow-up. Differences in outcome between groups were tested with the log-rank test. The prognostic relevance of LOX IR was examined by Cox regression analysis alone and together with GS. Probability of event-free survival (P-EFS) is presented ± standard error of the mean (SEM). An intraclass correlation analysis was used to validate the two different LOX scorings. The Mann-Whitney U test was used for comparison between hypoxic and control treated prostate cells, and for comparison between CRPC samples and non-malignant prostate/primary prostate tumors. The Wilcoxon Signed-ranks test was used for comparison between paired non-malignant prostate tissue and primary prostate tumors samples. The significance threshold was set at 0.05. Statistical analysis was performed using the SPSS 23.0 software (SPSS Inc., Chicago, USA).

## Results

### LOX expression in malignant and non-malignant human prostate tissues

**Fig 1:**
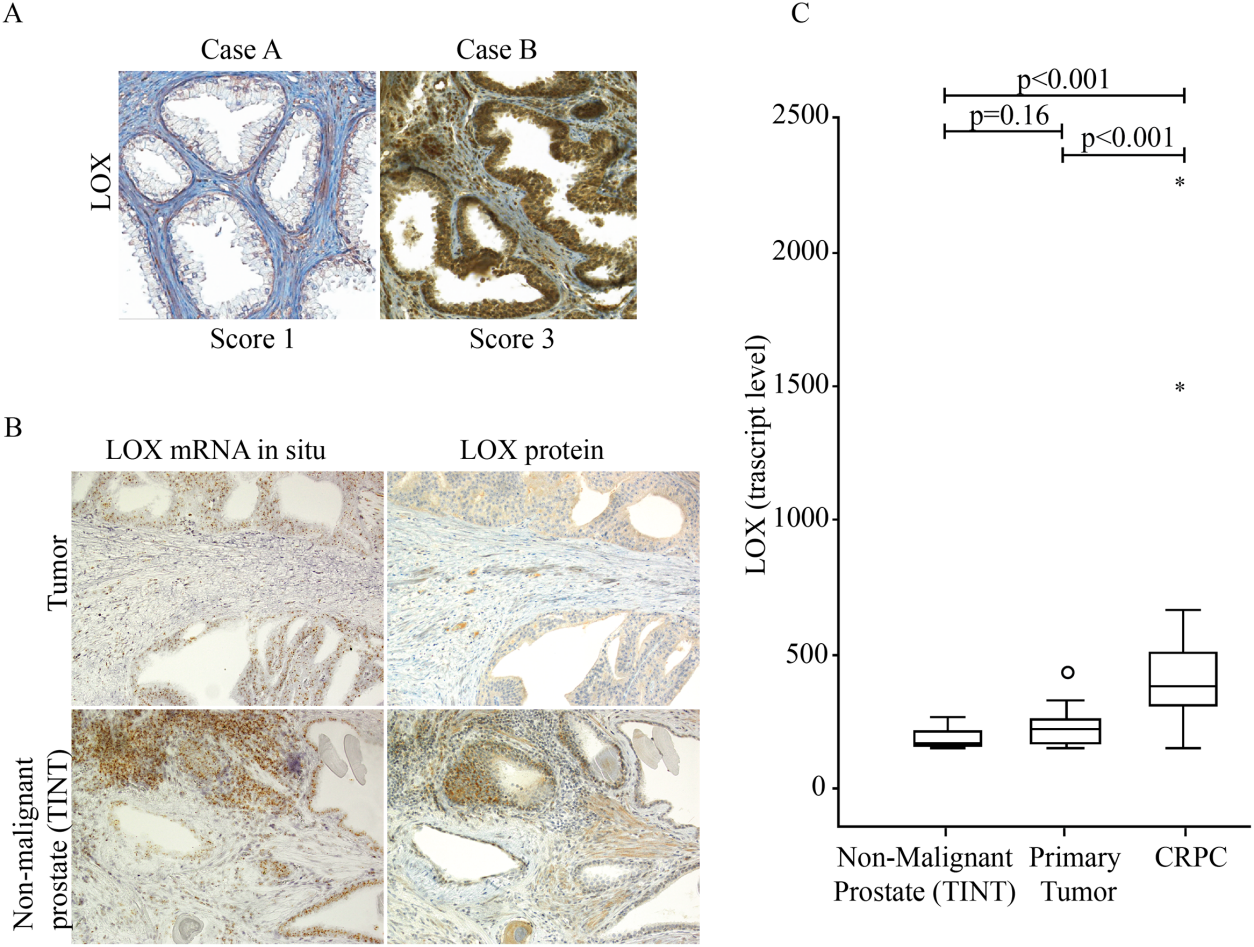
Lysyl Oxidase (LOX) expression in malignant and non-malignant human prostate tissue. (A) Representative immunohistochemical staining of LOX (brown) in sections of non-malignant prostate tissue specimens (TINT epithelium and TINT stroma) from two prostate cancer patients (original magnifications x 200). Case A show weak epithelial staining (score 1) and Case B strong epithelial staining (score 3). (B) Consecutive sections from non-malignant prostate tissue stained with *in situ* hybridization or immunohistochemistry for LOX. Note that mRNA (brown dots) and protein (brown) expression in individual glands were related, in the stroma in contrast mRNA was generally low but protein often detected (original magnifications x200). (C) LOX mRNA expression in non-malignant prostate tissue (TINT) (n = 12), in primary prostate tumor tissue (n = 12), and in castration-resistant bone metastases (CRPC) (n = 30) using Illumina gene expression array.

To evaluate the significance of LOX in prostate cancer, a TMA including tumor tissue and tumor-adjacent non-malignant prostate tissue (tissue cores were sampled at random distances from tumor tissue) from 351 prostate cancer patients with long follow-up was analyzed and scored by LOX immunohistochemistry in four different compartments; non-malignant prostate epithelium (TINT epithelium), non-malignant prostate stroma (TINT stroma), prostate tumor epithelium and prostate tumor stroma. In TINT epithelium a moderate LOX immune staining was generally observed in basal epithelial cells while luminal epithelial cell staining was variable between patients and scored from no or weak staining to moderate or strong (median score was 2, Fig 1A). LOX expression in TINT stroma was detected in smooth muscle cells and fibroblasts and staining intensity varied between patients from weak to strong (median score 2.5, Fig 1A). Tumor epithelial cells were generally positive and staining intensity in both tumor epithelium and tumor stroma varied between tumors (median score 2 for both sites, data not shown). LOX staining scores in the different compartments were all correlated to each other (data not shown). The strongest correlations were found between LOX score in TINT epithelium and the corresponding TINT stroma (*Rs = 0*.*50*, *p < 0*.*001)* and between tumor epithelium and the tumor stroma (*Rs = 0*.*44*, *p < 0*.*001*). To measure the consistency, two investigators scored LOX immune-reactivity independently and an intra-class correlation analysis gave a Chronbach’s alpha of 0.81 indicating a valid scoring.

To examine the location of LOX production in the prostate we used *in situ* hybridization. As the TMA material was too old for this assay, we examined and scored LOX immune staining and LOX mRNA expression index in 10 radical prostatectomy samples (< 3 year old material). LOX protein expression in the radical prostatectomies was similar to the staining in the in the TMAs showing variable staining intensity in both normal and tumor epithelial and stromal cells (Fig 1B). LOX mRNA was mainly expressed in the luminal prostate epithelial cells and in tumor cells with few positive cells in the non-malignant and malignant prostate stroma, showing that LOX was mainly produced in prostate epithelial and tumor cells (Fig 1B). Moreover, LOX mRNA expression index in epithelial cells correlated with the LOX staining score (*R*_*S*_ = *0*.*70*, *p < 0*.*05*).

We have previously examined the transcriptome of prostate tumor tissue in radical prostatectomies, in non-malignant prostate tissue in the same patients (sampled 0.5–3 cm from the nearest tumor foci, median distance 1 cm), and in castration-resistant (CRPC) bone metastases of separate patients [39, 40]. In these samples the transcript level of LOX mRNA was similar in non-malignant and malignant primary tumor tissue (Fig 1C). However, the LOX mRNA levels were significantly higher in CRPC bone metastases compared to the levels in the primary prostate tumors (Fig 1C). LOX mRNA levels in non-malignant prostate tissue appeared unrelated to distance from the tumor (data not shown) in line with results below demonstrating that LOX staining in TINT epithelium in the watchful-waiting cohort was only marginally related to tumor stage (and thus not closely relate to tumor size and average distance to nearest tumor).

### LOX immunoreactivity is associated with markers of tumor aggressiveness

Analyses were performed to investigate possible associations between LOX expression in the four different prostate tissue compartments to clinical parameters and histological characteristics of the tumor. LOX score in tumor epithelium was positively associated with GS, tumor stage, presence of metastases at diagnosis, tumor microvessel density and tumor proliferation (Table 1). LOX score in TINT epithelium was correlated to tumor stage, and tumor and non-malignant microvessel density (Table 1). However, LOX score in tumor stroma and TINT stroma appeared unrelated to these tumor characteristics (data not shown).

**Table 1:** Bivariate Correlations.

	Tumor epithelial LOX-IR		TINT epithelial LOX-IR	
	r	n	r	n
Gleason Score	0.33**	351	0.10	335
Local tumor stage	0.18**	345	0.11*	329
Metastases^†^	0.18**	279	0.05	263
Tumor microvessel density	0.21**	341	0.14*	317
Tumor cell proliferation (Ki67)	0.14**	345	0.09	320
Tumor cell pEGF-R	0.21**	263	0.19**	252
Tumor cell pAKT	0.34**	272	0.17**	241
Hyaluronan in tumor stroma	0.20**	346	0.03	323
TINT microvessel density	0.06	323	0.11*	324
TINT epithelial pEGF-R	0.25**	256	0.31**	256
TINT epithelial pAKT	0.13	214	0.29**	234
Hyaluronan in TINT stroma	0.11*	330	-0.04	332

Spearman’s rank and ^†^Kendall’s tau b correlation tests. Data used in the correlation analysis were collected at the time of prostate cancer diagnosis.

*p < 0.05,

**p < 0.001.

Abbreviations: IR, immunoreactivity; TINT, Tumor indicating normal tissue; r, correlation coefficient; n, number of patients.

In addition we examined if LOX score was associated with other factors detected in tumor and TINT tissue that relate to patient survival [6–9]. Tumor epithelial LOX score positively correlated to pEGFR IR both in tumor and TINT epithelium, as well as hyaluronan in tumor and TINT stroma. Tumor epithelial LOX score was also positively correlated to pAKT in TINT epithelium (Table 1). LOX IR in TINT epithelium was positively correlated to pEGFR and pAKT both in tumor and TINT epithelium (Table 1). This suggests novel relationships between LOX expression in the tumor and prostate TINT epithelium and these factors. Mast cells and S100A9 positive macrophages showed no correlation to LOX IR (data not shown).

### LOX immunoreactivity in prostate TINT epithelium predicts cancer specific survival

**Fig 2:**
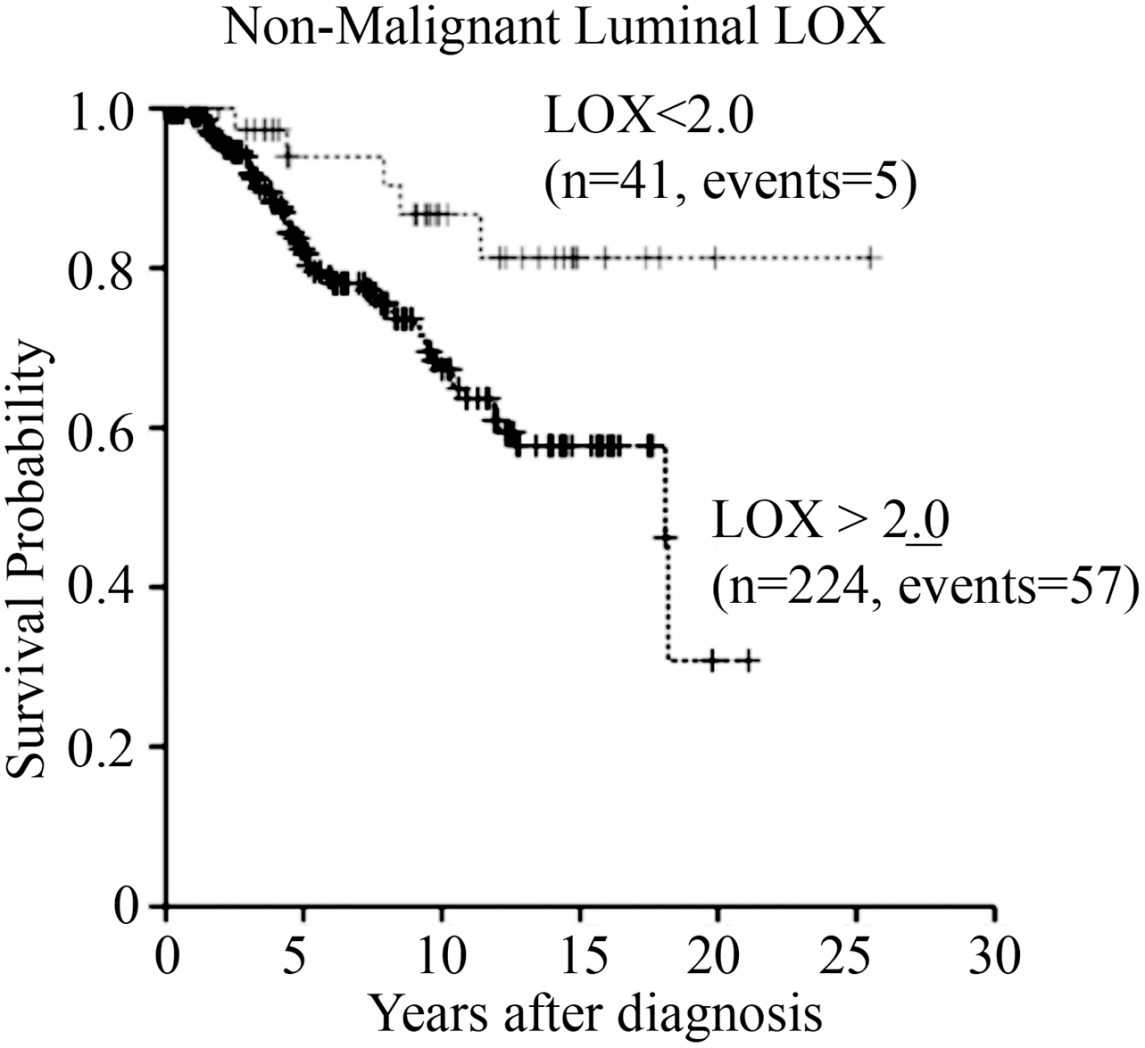
Survival curves. Patients divided into two groups depending on LOX immunoreactivity (IR) in non-malignant luminal epithelial prostate tissue (TINT epithelium).

Patients with high LOX expression (≥ the median value 2.0) in TINT epithelium had a significantly reduced cancer specific survival compared to the rest (15-year probability of event free survival (P-EFS) was 58 ± 5% and 81 ± 8% in the two groups; Fig 2). Furthermore, a high TINT epithelial LOX score was associated with an increased relative risk for prostate cancer specific death in a univariate Cox regression analysis (Table 2). In addition, LOX IR in TINT epithelium gave additive prognostic information to GS in a multivariate Cox regression analysis (Table 2). There were no significant relations between patient survival and LOX IR in tumor epithelium, tumor stroma or TINT stroma (data not shown).

**Table 2:** Cox regression for non-malignant luminal epithelial (TINT epithelial) LOX-IR in patients followed by watchful waiting.

Variable	n	RR	*P*-value	95% CI
(**A**) ***Univariate analysis***				
Gleason score**				
4–5	91	1*		
6–7	150	25.0	0.002	3.4–182.9
8–10	63	128.7	<0.001	17.6–939.5
TINT epithelial LOX**				
<2.0	41	1*		
≥2.0	224	2.9	0.024	1.2–7.2
**(B) *Multivariate analysis***				
Gleason score**				
4–5	83	1*		
6–7	130	24.4	0.002	3.3–179.4
8–10	52	104.5	<0.001	14.2–768.2
TINT epithelial LOX**				
<2.0	41	1*		
≥2.0	224	2.7	0.033	1.1–6.9

*Reference value.

**Cox regression analysis using Gleason score and TINT epithelial LOX-IR as categorical variables.

Abbreviations: n, number of patients; RR, relative risk; CI, confidence interval; IR, immunoreactivity.

### Hypoxia upregulates LOX in non-malignant prostate cells *in vitro*

**Fig 3:**
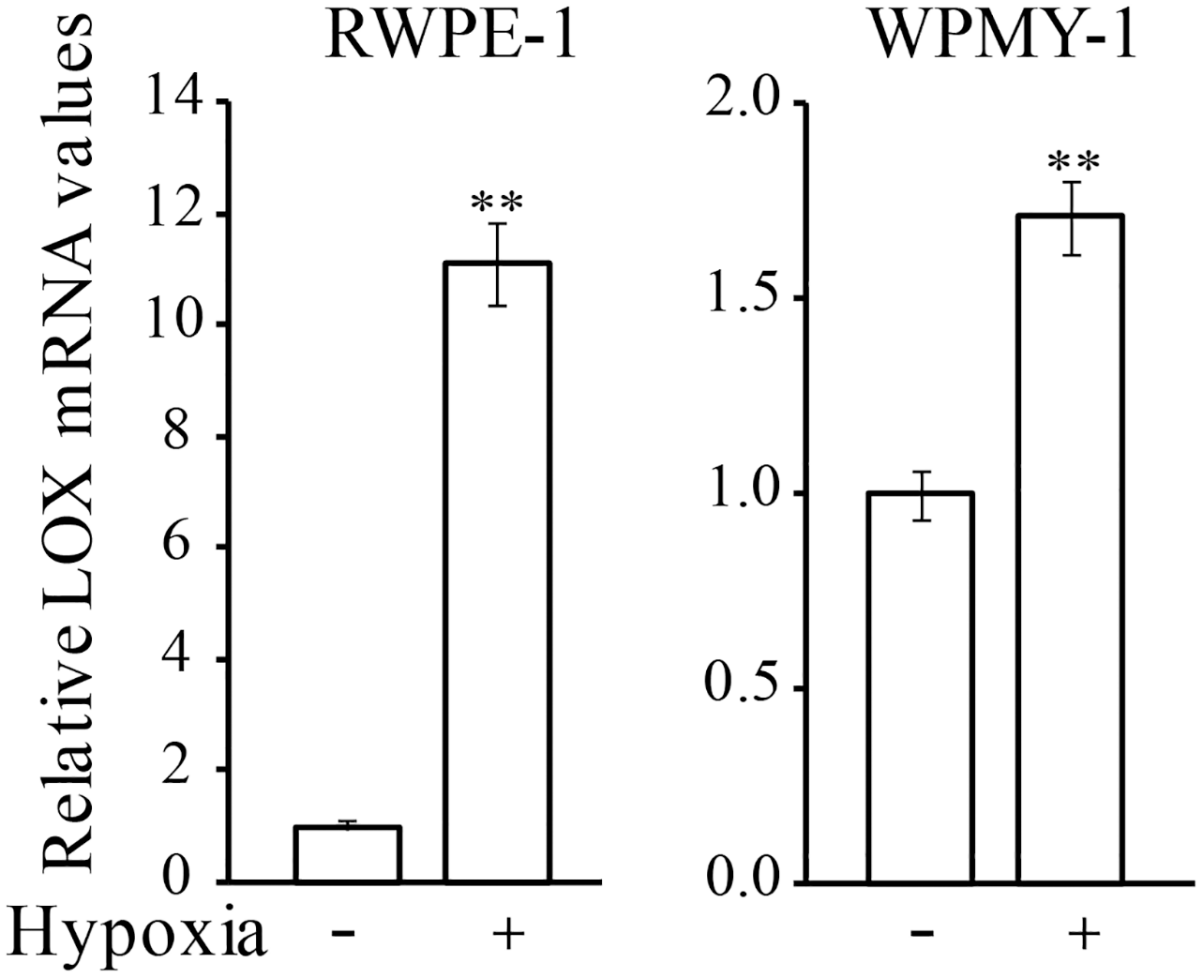
LOX mRNA expression in human prostate cells in hypoxia. Relative LOX mRNA expression in human prostate epithelial cells (RWPE-1) and human prostate stromal myofibroblasts (WPMY-1) grown *in vitro* in normoxic or hypoxic conditions for 24 h. Values are means ± SEM, **p < 0.01.

LOX expression has shown to be regulated by hypoxia [18, 41] and implantation of cancer cells induced hypoxia in the non-malignant parts of the tumor-bearing prostate lobe in rats [17]. Hypoxia could thus be one explanation for the high LOX expression seen in the non-malignant prostate epithelium of some prostate cancer patients as hypoxia is common in the epithelial compartments in non-malignant and malignant human prostate tissue [42]. In line with this we show that human prostate epithelial (RWPE-1) and stromal myofibroblast (WPMY-1) cells incubated in hypoxia for 24 hours *in vitro* had significantly higher LOX mRNA levels compared to normoxic controls (Fig 3). The magnitude of LOX up-regulation was highest in RWPE-1 cells (11-fold vs. 2-fold in WPMY-1). This suggests that hypoxia could up-regulate LOX expression in normal prostate epithelium and stroma.

## Discussion

Previous studies in a rat model suggested that the presence of a prostate cancer increased LOX expression not only in the tumor but also in the rest of the tumor bearing prostate lobe [17]. The aim of this study was therefore to explore if this could be the situation also in patients. This appeared to be the case and high levels of LOX in the tumor-adjacent non-malignant prostate epithelium (TINT epithelium) were associated with shorter cancer specific survival in prostate cancer patients. Furthermore, LOX score in prostate TINT epithelium gave additive prognostic information to GS. We have in animal models previously shown that adaptive changes occur in the surrounding tumor-bearing organ and that such changes can be used to prognosticate prostate cancers in patients [2, 5–10, 13]. Here we show that the LOX score in prostate TINT epithelium correlates with many factors previously examined in TINT epithelium (pEGFR, pAKT) and TINT stroma (microvessel density) and shown to relate to tumor aggressiveness and outcome [6–8, 10].

Serum PSA levels and Gleason scoring of tumor-containing biopsies has, for reasons outlined in the introduction, limited ability to predict tumor behavior in the majority of newly diagnosed cases [1]. Hence, additional diagnostic and prognostic markers, for example in the surrounding normal prostate tissue could therefore be potentially useful, particularly as non-malignant prostate is always sampled in the prostate biopsies [2, 4]. Our study suggests that LOX expression in the epithelium of the non-malignant part of the tumor-bearing organ could if validated in additional studies serve as an independent prognostic marker for prostate cancer. However, compared to pEGFR [7] and pAKT [6] LOX staining in TINT is apparently less informative.

Furthermore, and in line with some previous reports [34, 35] we show that LOX-IR in prostate tumor epithelium was correlated to several clinical characteristics related to tumor aggressiveness such as GS, tumor stage, microvessel density, tumor proliferation and metastases. Novel relationships between LOX expression in tumor epithelium and pEGFR, pAKT and hyaluronan were also found. Hyaluronan was recently suggested to work as an inducer of LOX expression in breast cancer cells, which may explain the positive correlation to LOX IR in tumor epithelial cells [43].

Ren et al. showed that LOX mRNA expression was predominantly expressed in normal human and mouse prostate epithelial cells, with lower levels expressed in the prostate stroma [36]. This and the finding in our *in situ* hybridization indicate that LOX is mainly produced by the prostate epithelium. Other gene expression studies have shown that LOX is expressed in both normal prostate epithelium and stroma [37]. In line with these studies we show that LOX protein was found in both human prostate non-malignant epithelium and stroma but that the levels of LOX varied between patients. One reason for the high LOX levels in prostate TINT could be that the tumor, by mechanisms unknown, induces LOX expression in non-malignant prostate cells. This is supported by our finding that orthotopic rat prostate tumor cells implanted to the prostate of rats resulted in increased expression of LOX mRNA in TINT compared to tumor-free control tissue [17].

We have recently shown that rat prostate TINT tissue gene-expression pattern is characterized by processes like inflammation, transforming Growth Factor-β (TGF-β) signaling and hypoxia [17]. Implantation of cancer cells into the rat prostate also induced hypoxia, as shown by pimonidazole staining, in the non-malignant parts of the tumor-bearing prostate lobe [17]. We here show that hypoxia significantly up-regulated LOX mRNA expression in human prostate epithelial cells and prostate myofibroblasts *in vitro*, and human prostate tissue (both tumor and surrounding normal) is generally hypoxic [42]. Hypoxia could thus explain the increased LOX levels in TINT and that increased levels of LOX is a consequence of tumor formation. A recent study by Cox et al. implicates that hypoxic breast tumors secretes LOX which subsequently induces pre-metastatic bone lesions [22]. Here, LOX score in tumor epithelial cells was found to be associated with bone metastases and LOX score in TINT epithelium to survival, suggesting that LOX secreted from the tumor and importantly also from the non- malignant parts of the tumor-bearing prostate could participate in pre-metastatic niche formation also in prostate cancer. Further studies are needed to examine this possibility and the relative importance of tumor vs. TINT secreted LOX. The hypothesis that the non-malignant parts of the tumor-bearing prostate could be a significant contributor is supported by our observation that LOX mRNA levels are similar in human prostate tumors and in adjacent non-malignant prostate tissue [39, 40].

TGF-β has been shown by others to upregulate LOX in prostate and breast tumor cells [36, 44] and aggressive prostate cancer cells expresses more TGF-β than indolent tumors and high TGF-β expression in tumor epithelial cells is associated with a poor outcome in our cohort of prostate cancer patients [45]. TGF-β stimulation of normal prostate epithelium could thus result in increased LOX expression and may explain some of the increased LOX in TINT. Furthermore, positive correlation of LOX with pEGFR and pAKT suggest that increased LOX expression is coupled to prostate epithelial cell activation. Further studies are needed to examine how prostate tumors may stimulate non-malignant prostate cells to increase their LOX expression.

This study suggests that high LOX synthesis in tumor epithelial and in the epithelial cells in the tumor-bearing organ may stimulate prostate tumor growth and spread. This finding is in line with several other studies listed in the introduction. Some studies, however, suggest that LOX could also be inhibitory and a gradual loss of LOX is seen in prostate cancer metastases [36] but not in the current one. In an experimental study we used a LOX-inhibitor (BAPN) in rats with implanted prostate tumors and found that LOX probably stimulated early tumor growth and conversely be inhibitory in already established tumors (Nilsson et al., unpublished). The functional role of LOX in prostate cancer could thus be context dependent.

One potential weakness of this study is the specificity of the LOX antibody used for immunohistochemistry. The antibody is raised against a synthetic peptide corresponding to an internal region of human LOX (within residues 200–300) and detects mature LOX and glycosylated variants on Western blot (www.novusbio.com). Although the staining pattern and intensity was in line with LOX in-situ hybridization, and with staining with an additional LOX antibodies (Sigma L4669 and Millipore ABT112, data not shown) we cannot exclude the possibility that our antibody may detect also other proteins, for example proteins in the LOX family (with similar functions as LOX). Another factor that needs consideration is that the current study is based on tissue obtained through trans-urethral resection of the prostate and the tumors could therefore be different from small cancers located only in the peripheral zone of the prostate. Future studies validating LOX staining should therefore examine it also in peripheral zone needle biopsies.

In summary, we show that high LOX expression in the tumor-adjacent non-malignant prostate epithelium (TINT epithelium) was associated with prostate cancer survival. LOX staining in prostate tumor epithelium positively correlated to Gleason score and metastases but was not associated with cancer survival.
